# A comparison of no-slip, stress-free and inviscid models of rapidly rotating fluid in a spherical shell

**DOI:** 10.1038/srep22812

**Published:** 2016-03-16

**Authors:** Philip W. Livermore, Lewis M. Bailey, Rainer Hollerbach

**Affiliations:** 1 School of Earth and Environment, University of Leeds, Leeds, LS2 9JT, UK; 2 School of Mathematics, University of Leeds, Leeds, LS2 9JT, UK

## Abstract

We investigate how the choice of either no-slip or stress-free boundary conditions affects numerical models of rapidly rotating flow in Earth’s core by computing solutions of the weakly-viscous magnetostrophic equations within a spherical shell, driven by a prescribed body force. For non-axisymmetric solutions, we show that models with either choice of boundary condition have thin boundary layers of depth *E*^1/2^, where *E* is the Ekman number, and a free-stream flow that converges to the formally inviscid solution. At Earth-like values of viscosity, the boundary layer thickness is approximately 1 m, for either choice of condition. In contrast, the axisymmetric flows depend crucially on the choice of boundary condition, in both their structure and magnitude (either *E*^−1/2^ or *E*^−1^). These very large zonal flows arise from requiring viscosity to balance residual axisymmetric torques. We demonstrate that switching the mechanical boundary conditions can cause a distinct change of structure of the flow, including a sign-change close to the equator, even at asymptotically low viscosity. Thus implementation of stress-free boundary conditions, compared with no-slip conditions, may yield qualitatively different dynamics in weakly-viscous magnetostrophic models of Earth’s core. We further show that convergence of the free-stream flow to its asymptotic structure requires *E* ≤ 10^−5^.

Numerical models of the geodynamo, the process by which the Earth’s magnetic field is sustained and generated in the Earth’s core, are now routinely computed by a variety of groups internationally. Although many of them produce Earth-like features, none can access the parameters describing the conditions representative of Earth’s core^1^. Two numerical parameters are of particular importance: the Rossby number, *R*_*o*_, a measure of the magnitude of fluid inertia, and the Ekman number, *E*, a measure of the magnitude of viscosity. Both of these non-dimensional numbers are believed small in Earth’s core, *Ro* ~ 10^−6^ − 10^−9^ (depending on the details of non-dimensionalisation) and *E* ~ 10^−15^, leading to a likely dominant magnetostrophic force balance between rotational, buoyancy, pressure and magnetic Lorentz forces in the bulk of the core. Typical values^2^ used in numerical models for these parameters are *R*_*o*_ ~ 10^−3^ and *E* ~ 10^−6^, highlighting the need for different modelling approaches to clarify behaviour at more realistic parameters.

The extreme smallness of the Ekman number in the Earth’s core means that viscosity is unlikely to play a dynamical role except in structures of small length scale. Examples of small scale structures include Stewartson layers, whose smallest lengthscale (in the cylindrical radial direction) is *O*(*E*^1/3^)^3^, and the pattern of convection rolls at onset, with lateral scales of *O*(*E*^1/3^) (see, for example the review in^4^). The focus of this study, however, is the class of very thin boundary layers in which viscosity rapidly alters the mainstream flow in order that it satisfies the physical no-slip conditions appropriate to the rigid boundaries defined by the solid inner core and overlying mantle (here assumed spherical). Away from the equator, the depth of these boundary layers scales as *E*^1/2^, giving an Earth-like value of approximately 1 m thick, about a million times smaller than the radial depth of the fluid core, well beyond the reach of state-of-the-art time-dependent 3D numerical models. Close to the intersection of the equator with the inner core, the boundary layer depth scaling alters to *E*^2/5^ 3, a property anticipated (although still unproven) to hold close to the intersection of the equator and the outer edge of the core. Within the bulk of the core, away from boundary layers, the fluid is expected to evolve according to inviscid dynamics.

Although encompassing only a tiny fraction of the core volume, these boundary layers are an unavoidable yet important feature in any geodynamo model: at the very least, the fine spatial scales needed to resolve them require considerable computational resources. If the boundary layers do nothing more than supply a matching condition for the main-stream flow, they are termed *passive*, and may be viewed as simply a numerical nuisance for the modeller. On the other hand, because the core is rapidly rotating, the boundary layers may well be *active* in the core, a well known example of which is the action of Ekman pumping, the movement of fluid into and out of the boundary layers and the formation of a secondary circulation of magnitude *E*^1/2^ 5,6.

Examples of active Ekman layers can be found within many fluid dynamical contexts in which rotation is dominant. In the laboratory for example, where the Ekman number is large compared with the core (*E* ≈ 10^−4^), Ekman pumping explains the rapid meridional circulation of angular momentum throughout the fluid, and therefore its efficient spin-up and spin-down. In the oceans where the Ekman number is small (*E* ≈ 10^−8^) but still much larger than core-like values, Ekman pumping drives flows important in the formation of ocean gyres and in coastal upwelling. Possibly the best analogue for the core, however, is the atmosphere in which the Ekman number is directly comparable, typical estimates being *E* ≈ 10^−13^. Although not important for determination of the bulk flow, boundary layers are important and indeed simple Ekman effects qualitatively explain the up-flow in low pressure regions (leading to clouds and rain) and down-flow in high pressure regions (leading to clear skies)^7^.

Within Earth’s core, boundary layer effects may well also be important; indeed, Ekman pumping has already been identified^8^ in the development of convection columns in geodynamo models (albeit at very high Ekman numbers of 10^−4^). In Earth’s core, the comparative magnitude of the Ekman number would mean that any such pumping would be significantly less than in these models although it may remain important. A further boundary effect, relevant to Earth’s core, arises due to its spherical geometry and the presumed magnetostrophic balance. In the absence of inertia, any non-zero axially averaged torque (i.e. if the body forces do not satisfy the constraint of Taylor^9^) must be balanced by viscous drag of large-scale flows within the boundary layer. Because of the smallness of the Ekman number, these flows have a structure and a (very large) magnitude that is completely controlled by the dynamics of the boundary layer. Such boundary-induced flows are a principal feature of the weakly-viscous magnetostophic regime, and indeed control the time-step of computational models^10^.

The goal of geodynamo modellers is to accurately describe the dynamics of rapidly rotating convection and its role in magnetic field generation. In their attempts to achieve this, some modellers have aimed to simplify the spatial description of boundary dynamics, freeing up valuable computational resources that can used, for example, to extend the model run-time. On the basis that the extreme smallness of the Ekman number in the core makes viscous coupling between the core and mantle negligible, at least on centennial-millenial timescales on which the magnetic field is generated, in some models a zero-tangential stress condition in place of a no-slip condition has been adopted^11^,^12^,^13^,^14^. Because the stress-free condition allows non-zero flow at the boundary, the free-stream flow therefore requires much less adjustment at the boundary, and the role of the boundary layer is consequently reduced.

Yet it remains unclear whether or not this change in mechanical boundary condition, significantly altering the dynamics of the boundary layers, will alter the global properties of the free-stream flow and therefore the ability of the computations to model accurately the dynamical equations. Due to reduction in viscous-drag at the boundaries, stress-free calculations typically give much larger zonal flow velocities than their no-slip counterparts^15^,^16^, indicating that the solutions depend in a fundamental way on the boundary conditions, at least within the currently accessible parameter regime. Furthermore, the structure of the most unstable mode at laminar onset of (non-magnetic) thermal convection, highly relevant here because the geodynamo is driven by convection, depends in a fundamental way on the choice of velocity boundary condition^17^; in fact the dependence of the critical Rayleigh number (measuring the convective driving) as a function of Ekman number at low Prandtl number (as relevant to planetary cores) is fundamentally different between no-slip and stress-free conditions^18^. Additionally, even in fully developed (non-magnetic) convective turbulence, a recent study^19^ showed that despite the magnitude of the Ekman flows being comparatively small (and decreasing with *E*), their effect on the heat transport (measured by the Nusselt number) depended in a fundamental way on the choice of boundary conditions.

The dynamical role of the boundary layers at low-*E* in Earth’s core appears therefore to be an open question, the uncertainty due to the inaccessibility of realistic values of viscosity in computer models. The purpose of this paper is to investigate the role of both no-slip or stress-free boundary conditions at Ekman numbers not only many orders of magnitude smaller than those possible in full simulations, but at asymptotically low *E*. This is possible by simplifying the modelled dynamics, retaining only those terms believed important for a magnetostrophic balance in the core. Because non-axisymmetric and axisymmetric solutions behave very differently, we have divided our analysis into two. Our first goal, within the confines of non-axisymmetric solutions, is to investigate the structure of the boundary layers and model convergence in the limit *E* → 0 to the inviscid case *E* = 0. We are able to quantitatively confirm convergence of the weakly-viscous free-stream solution to the formally inviscid solution outside of the boundary layers. Our second aim is to investigate the structure of the axisymmetric flow; as we will show, of particular interest is the scaling of the zonal flow driven by viscous effects and its apparent crucial dependence on the boundary conditions. We end the manuscript with a discussion.

## Boundary Layer Structure

### The model and numerical method

The basis of our model is the inertia-less, non-dimensional form of the Navier-Stokes equation for the incompressible flow **u**:


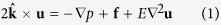


where 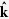 is the unit vector in the direction of rotation, *p* is the pressure, and **f** is a body force (in the case of the geodynamo, the sum of the Lorentz and buoyancy forces). Following a standard approach^20^, we have chosen scales for length as the core radius *L* = 3480 km, time as the magnetic diffusion time *T* = *L*^2^/*η* where *η* ~ 1 m^2^/s is the magnetic diffusivity and the flow velocity as *L*/*T*. We have neglected the inertial terms on account of the small magnitude of the Rossby number in Earth’s core. In some of our models we retain viscosity in order to investigate the boundary layers, but we also are able to construct inviscid models in which there are no boundary layers, providing a benchmark of convergence within the free-stream. The inviscid (and inertia-free) equations have no solution unless Taylor’s constraint^9^ is satisfied, which we do here by choosing **f** to have a single azimuthal wavenumber dependence *m* = 1^21^. By neglecting inertia, our kinematic prescription of **f** drives a flow which is assumed steady; any (time-dependent) dynamic adjustment of **f** is ignored in this study.

We solve the weakly-viscous and inviscid equations in the spherical-shell domain *r*_*i*_ ≤ *r* ≤ *r*_*o*_ where (*r*, *θ*, *ϕ*) are spherical polar coordinates, *r*_*i*_ = 1/2 and *r*_*o*_ = 3/2 representing the inner and outer core boundary radii respectively. For the viscous models, we note that the Ekman number 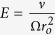 is based on the core viscosity *ν*, the Earth’s rotation rate Ω and the outer-core radius *r*_*o*_, which we take to have values in the range [10^−3^, 10^−9^]. Although we cannot approach Earth-like extremes for this parameter, we anticipate that we can extrapolate using scaling arguments provided that the asymptotic low-*E* regime has been reached.

The weakly-viscous cases must be solved numerically, for which the flow **u** is decomposed into toroidal (*e*) and poloidal (*f*) scalar functions. It is computationally expedient to choose **f**, not only to have single wavenumber dependence, but also to be equatorially symmetric (*E*^*S*^): the flow has the same symmetry and can be written as the real part of


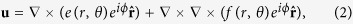


where *e* (*f*) are now equatorially antisymmetric (symmetric) scalar functions, represented in terms of spherical harmonics and Chebyshev polynomials^22^, and 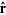 is the unit position vector. Projecting the radial components of both the curl and of the double curl of (1) onto spherical harmonics and a radial grid yields linear equations for the modal coefficients. We impose either no-slip and stress-free boundary conditions, whose form is respectively


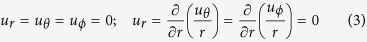


which can be readily converted into constraints on the spherical harmonic components of *e* and *f* (e.g.^23^). The resolution adopted is 400 spherical harmonic modes (of the correct equatorial symmetry, i.e. up to degree 800 for fixed order) and 400 Chebyshev polynomials for *E* ≥ 10^−7^; higher resolution is used where needed for smaller Ekman numbers. It is worth noting that the inviscid solutions to (1) satisfy only the no-penetration condition *u*_*r*_ = 0 at *r* = *r*_*i*_, *r*_*o*_.

In order to minimise structures on and around the tangent cylinder 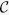 (the cylinder of fluid parallel with Earth’s rotation axis and tangent to the inner core) which are not the focus of this study, we employ the *m* = 1, *E*^*S*^ body force^24^ with parameters *A*_0_ = −53/4, *A*_1_ = 25, which is of the form


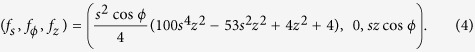


This body force drives a flow which is not only continuous but has finite derivatives across 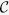. For this choice of forcing, following the method of Livermore & Hollerbach^24^, it is straightforward to calculate the exact analytic inviscid solution that satisfies (1) with *E* = 0 and the single boundary condition *u*_*r*_ = 0. Outside the tangent cylinder, *s* ≥ *r*_*i*_:


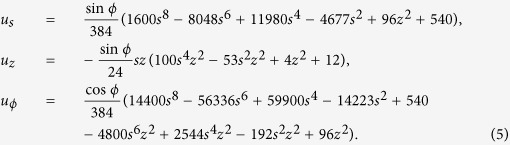


Similar but more lengthy analytic expressions for the flow inside the tangent cylinder exist, but they are omitted here. In this paper, we restrict ourselves to reporting equatorially symmetric solutions, although we have computed analogous equatorially antisymmetric solutions and find the results to be similar.

Lastly, we shall visualise the models by plotting profiles in radius through the boundary layers. Although these will depend on azimuthal angle, we can strip away the known azimuthal dependence in the limit *E* → 0, following^24^, by considering only the component of flow given by the tilde-variables in


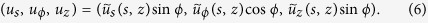


We shall henceforth drop the tilde superscript when the context is clear.

### Boundary layer structures and scalings

**Figure 1:**
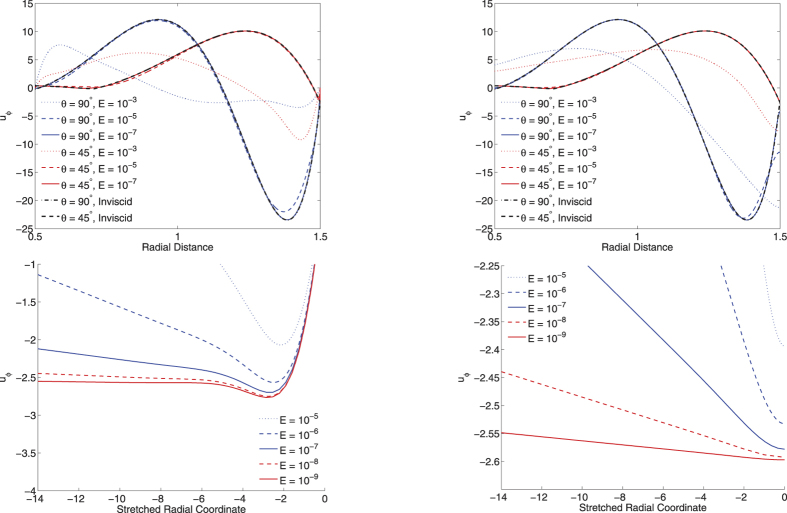
Top row: the *u*_*ϕ*_ component along radial profiles at colatitude 90° and 45°, for various Ekman numbers *E* = 10^−3^, 10^−5^, 10^−7^, with both no-slip (left) and stress-free (right) boundary conditions applied. Second row: similar profiles but now along the stretched coordinate *r*′ = *E*^−1/2^(*r* − 3/2) at colatitude 45°. In the top row, *E* = 10^−9^ is omitted for graphical reasons.

Even prior to any further boundary-layer analysis, simple plots of the velocity components provide insight into the effect of the different boundary conditions. The top row of Fig. 1 shows the radial profiles of *u*_*ϕ*_ at colatitudes of 45° and 90° that intersect perpendicularly the outer boundary. A first observation is that the solutions divide into a main-stream solution which is brought rapidly to zero by boundary layers, whose depth decreases with *E*. In the bulk of the domain, models with both no-slip and stress-free solutions converge to the inviscid solution showing that, in this particular case, the choice of boundary condition does not impact the structure of the mainstream flow. Of the two choices, models with stress-free conditions converge faster to the inviscid solution. It is worth remarking that both choices of boundary conditions require a boundary layer, since the inviscid solution satisfies none of the tangential conditions in (3). A second observation is that values of *E* = 10^−5^ present only marginal convergence to the inviscid solution, values of *E* = 10^−3^ give solutions very far from the low-E limit. Lastly, it is worth remarking that the stress-free boundary conditions do not constrain the value of the velocity itself at the boundary. Thus all values of *E* give models with different values of *u*_*ϕ*_ at *r* = *r*_*o*_, the difference between the solutions being largest at the boundary.

Anticipating that the structure inside the boundary layers scales as *E*^1/2^, the second row of Fig. 1 shows profiles of *u*_*ϕ*_ along the stretched coordinate *r*′ = *E*^−1/2^(*r* − *r*_*o*_) at colatitude 45°. In the no-slip case, we can identify convergence to a structure independent of *E* for |*r*′| ≤ 3, demonstrating that the boundary-layer depth scales as *AE*^1/2^ with *A* ≈ 3. The stress-free case is less clear, due to the fact that *u*_*ϕ*_(*r*_*o*_) is not constrained to any particular value, but shows that the curves have the same gradient in the range [−1, 0], indicating that the boundary layer depth scales as *BE*^1/2^ with *B* ≈ 1.

Finally, we remark that of the three velocity components, boundary layer structures are only visible in the tangential components, *u*_*θ*_ and *u*_*ϕ*_. This is because the spherical radial component *u*_*r*_ satisfies the same boundary condition in all of the no-slip, stress-free or inviscid cases, and so the weakly viscous solutions require no adjustment in the value of *u*_*r*_ close to the boundary to match the inviscid solution.

Having demonstrated that the boundary layer depth follows the expected scaling, we now adopt a more empirical approach and investigate whether we find the same scalings when we define the boundary layers to be the locations where the viscous forces become important in comparison to the Coriolis term. Along radial profiles, we evaluated the ratio of the numerically computed viscous term at non-zero *E*, normalised by the Coriolis force for the exact *E* = 0 solution given by (5):


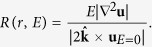


The closest local maximum of *R* to *r* = *r*_*o*_, *R*_*max*_, defines the heart of the boundary layer where viscosity is very important. The ratio *R* falls off rapidly as *r* decreases, and we defined the depth of the boundary layer, *δ*, as the depth below *r*_0_ at which *R* had decreased by a factor of 10, that is, *R*(*r*_*o*_ − *δ*) = 0.1*R*_*max*_.

**Figure 2:**
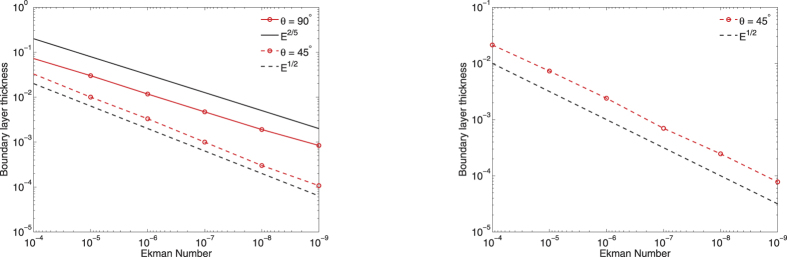
The scaling with Ekman number of the thickness of the boundary layer *δ*, for no-slip (left) and stress-free (right) boundary conditions.

Figure 2 shows the non-dimensional thickness of the boundary layer as a function of Ekman number. For both choices of boundary condition, away from the equator, the depth scales as *CE*^1/2^ where the constant *C* is about 2 in the no-slip case and about 3 in the stress-free case. On the equator, for no-slip conditions the boundary layer depth thickens to *E*^2/5^; for stress-free conditions, there is no easily quantifiable boundary layer depth. Thus the two methods of characterising boundary layer depth presented, either inspecting profiles of *u*_*ϕ*_ or by assessing where viscosity is dominant, agree very well. When scaled to dimensional values, at *E* = 10^−15^, with no-slip conditions the boundary layer depth is about 0.2 m, compared to 0.1 m with stress-free boundaries. The reduced thickness of the boundary layer in the stress-free case, compared to the no-slip case, is simply a reflection of its relatively reduced role.

**Figure 3:**
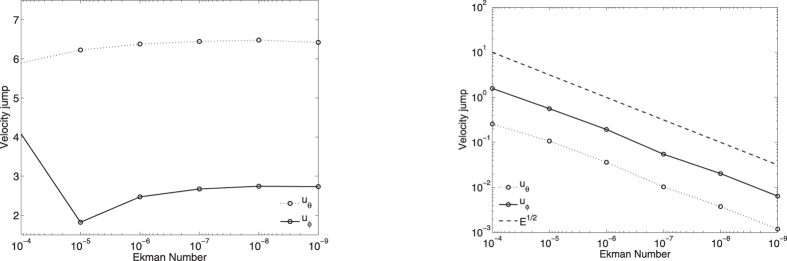
The dependence on Ekman number of the jump in *u*_*θ*_ and *u*_*ϕ*_ across the boundary layer [*r*_*o*_ − *δ*, *r*_*o*_], for no-slip (left) and stress-free (right) boundary conditions at colatitude 45°. In the stress-free case, the scaling of *E*^1/2^ is shown for comparison.

Another important aspect of the boundary layers is the velocity jump that occurs across them. Figure 3 shows the jump, between *r*_0_ − *δ* and *r* = *r*_*o*_, in both tangential flow components along spherical radial profiles at colatitude 45°. In the no-slip case, the jump tends to a finite value, being the difference between the inviscid solution at *r* = *r*_*o*_ (which is non-zero) and the viscous tangential flow there (which is zero). In the stress-free case, the velocity jump scales as *E*^1/2^.

**Figure 4:**
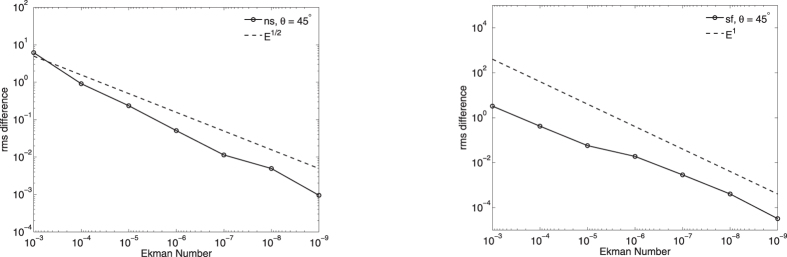
Convergence (in rms) of the weakly-viscous profiles of *u*_*ϕ*_ to the inviscid solution away from the boundaries and the tangent cylinder, for no-slip (left) and stress-free (right) boundary conditions at colatitude 45°. In each case, scalings of *E*^1/2^ and *E*^1^ are shown.

Lastly, we turn attention away from the boundary layers to the main-stream flow. Figure 4 shows the rate of convergence of the numerical weakly-viscous solutions to the analytic inviscid solution within the bulk of the domain at colatitude 45°, which we measure using the rms quantity Ψ, where


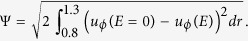


The value of Ψ is computed using trapezoidal quadrature (with a large number of points, sufficient for convergence), and is taken over a region well away from tangent cylinder (which is at spherical radius 0.7 at this colatitude) or boundary layer effects. The plots show that the deviation from the inviscid solution scales as *E*^1/2^ in the no-slip case and as *E*^1^ for stress-free, in accordance with the anticipated asymptotics^25^.

## The Geostrophic Flow

### The model and numerical method

In the previous section we demonstrated that weakly-viscous non-axisymmetric solutions, subject to either boundary condition, converge to the inviscid solution away from the boundary layers. For this geometry of solution, the inviscid flow exists because the body force (recall that it is of single azimuthal wavenumber *m* > 0) trivially satisfies Taylor’s constraint^9^, a statement about the average torque:


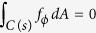


where *C*(*s*) is a cylinder of fluid of cylindrical radius *s*, coaxial with the rotation axis, and *dA* is the surface element. The role of viscosity is then simply to alter the main-stream flow within boundary layers in order to satisfy the required boundary conditions. Although it is far more difficult for the axisymmetric component of **f** to satisfy Taylor’s constraint, when this does happen, the axisymmetric flow will have comparable boundary effects as the non-axisymmetric case.

However, when **f** violates Taylor’s condition, the inviscid solution to (1) no longer exists. Instead, in the absence of inertia, only viscosity can act to balance the torque, which depends on the thickness of the boundary layer and the structure of the velocity drop across it, both of which depend on the choice of boundary condition imposed. For any given required viscous torque, weaker viscosities require a larger velocity drop, associated with stronger zonal flows.

In this section, we investigate the impact of the choice of boundary condition on the axisymmetric flow within weakly-viscous magnetostrophic models. This component of flow can be separated into two components: its geostrophic component, *u*_*g*_, the azimuthal component of flow averaged over cylinders *C*(*s*), and the residual ageostrophic component. At small Ekman numbers, the geostrophic component dominates the zonal flow and it is only this which we investigate here. It is noteworthy that, when *E* = 0, *u*_*g*_ is not constrained by (1) but is instead determined through considerations of the time-independence of Taylor’s constraint: a completely different prescription than solving (1) directly. Although analytic formulae have been derived pertaining to either no-slip and stress-free boundary conditions for the structure of *u*_*g*_ for small but non-zero *E*, they appear never to have been compared in any specific case; this is our focus here.

We assume that the body force, **f**, comprises only the magnetic Lorentz force with no buoyancy; this is purely for simplicity and for ease of comparison to the previous literature. The background magnetic field is defined as


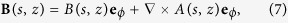


where *B* and *A* are toroidal and poloidal scalars, respectively, and where **B** satisfies an electrically insulating matching condition at *r* = *r*_*o*_ (e.g.^12^).

Asymptotic theory predicts that, in the no-slip full-sphere case,


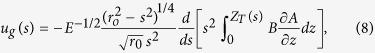


whereas for stress-free boundaries


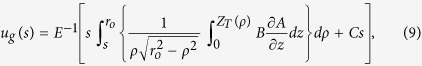


where 
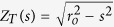
 is the half-height of a cylinder, (*s*, *ϕ*, *z*) are cylindrical coordinates and the constant *C* is a free parameter. We note an apparent sign error in this formula given in^25^, although the original derivation in^26^ remains correct. We have changed the range of integration, originally over [0, *s*], to [*s*, *r*_*o*_], in order that this formula is valid outside the tangent cylinder in a spherical shell. Equations (8) and (9) are usually quoted for a sphere of unit radius, although formulae valid for a sphere of radius *r*_*o*_ are immediate on rescaling all variables containing a length scale (including the Ekman number and the geostrophic flow), and recalling that time is defined relative to magnetic diffusion time which scales as the square of length. In a full sphere study, we may choose *C* so that the solution has zero angular momentum, but here we choose *C* such that the asymptotic and numerical solution at *E* = 10^−8^ agree at *s* = 1. It is interesting to remark that not only do these formulae scale differently: *E*^−1/2^ compared with *E*^−1^, but that they multiply a spatial structure whose profile may be different.

The details of how we select *A* and *B* are given here. We choose the magnetic field **B** to be (i) equatorially symmetric, (ii) to violate Taylor’s constraint (see below), (iii) to drive a continuous *u*_*s*_ across 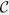, (iv) to be regular everywhere (v) to be large-scale. We choose the poloidal, *A*, and toroidal, *B*, scalars in a spherical geometry to be of spherical harmonic degree 1 and 2 dependence respectively, and with radial structure defined in terms of the polynomial Galerkin basis described in^27^,^28^, each member of which satisfies the required insulating condition and is regular at the origin. The difficulties inherent in the fact that *u*_*g*_ is most naturally calculated in cylindrical components, yet the magnetic field satisfies a condition in spherical polar coordinates, are circumvented because the Galerkin scheme guarantees **B** has a simple regular polynomial representation in both coordinate systems. The toroidal radial structure is chosen to be that of the largest scale mode, and the poloidal structure a linear combination of the largest two scale modes, the single degree of freedom sufficient to satisfy the condition of^29^, ensuring that *u*_*s*_ is at least continuous across 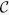. The poloidal and toroidal scalar functions are then:


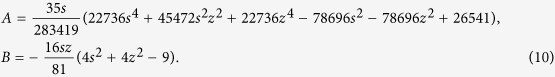


The same spherical code as described earlier was run (in axisymmetric mode) using this new structure for ***f***. The geostrophic flow was then determined by computing the cylindrical average of *u*_*ϕ*_(*r*, *θ*), using Gauss quadrature in the *z*-coordinate (with a sufficiently large number of abscissae).

In our method, we have deliberately chosen a Lorentz force that does not satisfy Taylor’s constraint, here of the form


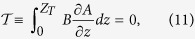


otherwise *u*_*g*_ would be zero in both (8) and (9). It is clear that both formulae break down in the inviscid limit *E* = 0, when *u*_*g*_ must be determined by the method of Taylor^9^.

Lastly, it is worth remarking that some of our no-slip calculations are directly comparable to the work of Liao & Zhang^30^, who studied solutions of the same equation at low *E* using a different body force that also did not satisfy Taylor’s constraint. They confirmed agreement between an asymptotic method for the full problem (not just the geostrophic part) and their numerical method at Ekman numbers no smaller than *E* = 10^−5^. Interestingly, the *E*^−1/2^ scaling of the geostrophic flow (and therefore a scaling of *E*^−1^ of the kinetic energy) was not apparent in their results, presumably because *E* was insufficiently small for the geostrophic flow to dominate.

### The structure and scalings of the geostrophic flow

**Figure 5:**
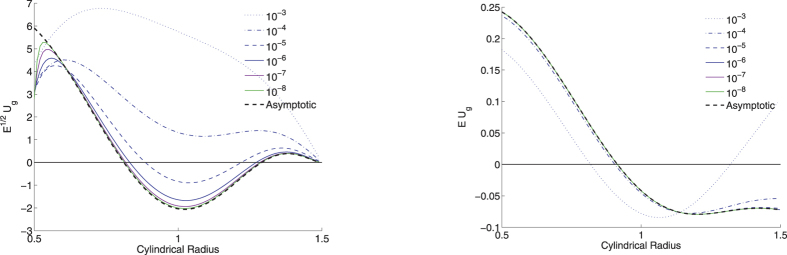
Profiles of *u*_*g*_ scaled by *E*^1/2^ and *E* for no-slip (left) boundary conditions and stress-free (right) boundary conditions respectively. In all cases, the numerical solution converges to the analytic asymptotic profile although more rapidly in the stress-free case.

Figure 5 shows profiles of the geostrophic flow (with the leading-order Ekman number scaling removed), *E*^1/2^*u*_*g*_(*s*) and *Eu*_*g*_(*s*), for no-slip and stress-free boundary conditions respectively. In both cases, the numerical solutions converge to the asymptotic formulae, an important check that confirms both the analytic and numerical approaches. As previously identified for boundary layers, the stress-free case converges more rapidly in *E* to the asymptotic structure than the no-slip case. In the no-slip case, we require *E* ≤ 10^−6^ to get reasonable agreement between the weakly-viscous and asymptotic solutions; in the stress-free case *E* ≤ 10^−5^ is sufficient.

The complications arising from the tangent-cylinder (*s* = 1/2) are particularly evident for the no-slip boundary condition, in whose vicinity the numerical solutions deviate from the full-sphere asymptotic case. This arises in spite of the fact that we have chosen **f** judiciously in order to make *u*_*s*_ continuous across 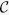. The generalisation of (8) inside the tangent cylinder (following an analogous treatment to that in^31^) is


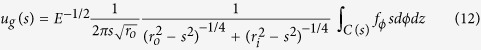


which vanishes as *s* → *r*_*i*_ from below due to the infinite slope of the inner-boundary. Here, the integral term in *f*_*ϕ*_ is a generalisation of the integral terms in (8). Thus the no-slip conditions on the inner core have a fundamental effect on the low-E solutions; by comparison, for stress-free boundaries the deviation between full-sphere and spherical shell appears negligible outside the tangent cylinder.

Perhaps the most crucial observation however is that the two asymptotic profiles corresponding to the different boundary conditions are not the same in this example; although both are positive near the tangent cylinder, they differ in sign towards the outer boundary: one has eastward and one westward drift near the equator. Thus the choice of boundary condition has a fundamental effect on both the scaling and spatial structure of the geostrophic flow.

**Figure 6:**
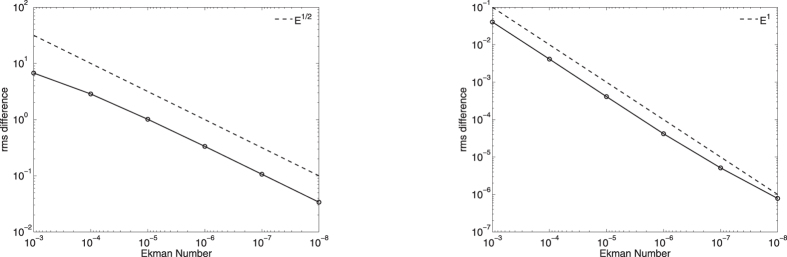
The dependence on Ekman number of the scaled rms difference in *u*_*g*_ between the weakly-viscous and the asymptotic profiles, for no-slip (left) and stress-free (right) boundary conditions. In each case, scalings of *E*^1/2^ and *E*^1^ are shown.

Finally, Fig. 6 quantifies the rate of convergence (in *E*, away from the boundaries) of the numerical to the asymptotic solutions by an rms measure:


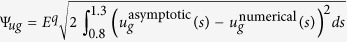


where *q* = 1/2 in the no-slip case, and *q* = 1 in the stress-free case. For these two cases, the no-slip rms scales as *E*^1/2^ whereas the stress-free rms scales as *E*^1^, consistent with the asymptotic corrections to the geostrophic flows for both no-slip and stress-free being *E*^0^.

## Discussion

In this paper we have investigated the role of the choice of mechanical boundary condition on the boundary layers, free-stream and zonal flows in weakly-viscous numerical solutions. We confirmed that, irrespective of boundary condition, non-axisymmetric solutions converge to the free-stream (inviscid) flow outside thin boundary layers. These boundary layers scale generally as *CE*^1/2^, where *C* is an empirically determined constant of magnitude *O*(1), giving dimensional values in Earth’s core (assuming *E* = 10^−15^) of either approximately 0.2 m or 0.1 m for the no-slip and stress-free cases respectively. If turbulent values of viscosity are adopted, leading to greater Ekman numbers of *E* = 10^−9^, these thickness alter to 200 m or 70 m, respectively. It is significant to note, however, that although the role of the boundary layer may well be reduced in the stress-free case, approximately the same radial resolution will be needed in a numerical model to resolve the boundary layers for either choice of condition; the computational saving in using stress-free conditions rather than no-slip, one of its primary motivations, may therefore not be as great as is commonly thought. In the presence of an exact axial torque balance (i.e. where Taylor’s constraint is satisfied), both axisymmetric and non-axisymmetric solutions would have comparable Ekman flows. Although *E* is very small, these secondary flows may still be important in core dynamics, either by influencing large-scale structure or through their effect on transport properties such as heat-transfer at the boundary.

The numerical resolution of Ekman boundary effects and associated secondary flows often comes at a high computational cost, particularly at low Ekman number. In some geometries, for example Cartesian^19^ and spherical quasi-geostrophic^32^, it is possible to build into the modelling a parametrisation of Ekman pumping, which then removes the need to resolve its dynamics explicitly. In a sphere, such a parameterisation is given by equation A2 of Schaeffer *et al.*^33^, which is shown not only to be discontinuous but singular (with *u*_*r*_ ~ (cos*θ*)^−3/2^) at the equator. Because the Ekman layer breaks down in the vicinity of the equator, the singularity of such a description is not surprising. Apparently, within the QG framework^33^, the singularity (which remains) is not strong enough to affect the large-scale numerical solutions. In a Cartesian geometry, there is no singularity and in fact the Ekman effects are well represented by this parameterisation^19^.

The axisymmetric solutions are a special class of flow, because they contain the geostrophic flows in a sphere, whose sole (azimuthal) component depends only on cylindrical radius. As we have shown, if viscosity is required to balance any non-zero axial torque, these zonal flows have not only a structure but a scaling that depends fundamentally on the choice of boundary condition. A no-slip boundary condition requires a zonal flow of magnitude *E*^−1/2^, whereas the weakened effect of viscosity in the stress-free case requires a zonal flow of magnitude *E*^−1^ to create the required viscous drag.

It is noteworthy that our computed geostrophic flows, when converted to dimensional units, give unrealistically large magnitudes: 100 m/s and 10^8^ m/s for the no-slip and stress-free cases, respectively, compared to typical flow velocities inferred inside the core of 10^−4^ m/s^34^. These excessive values are due entirely to the fact that, for our example magnetic field, Taylor’s constraint is not close to being satisfied: 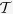 has an rms value of about 3 over 1/2 ≤ *s* ≤ 3/2. If we assume the magnitude of core-surface flow is typical of flows within the core, then this requires 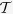 to be a factor of 10^6^ or 10^12^ smaller to match observations. Assuming further that this occurs by internal cancellation of magnetic torque, rather than by changing the magnitude of the field itself (assumed to be a few mT), it then follows that, in order to approximate the core by a weakly-viscous magnetostrophic model, the cancellation inherent within Taylor’s constraint (given by 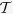 = 0) needs to be satisfied to approximately one part in 10^6^ for no-slip models, and in stress-free models to no more than one part in 10^12^. This then provides some idea of the accuracy needed for numerically solving the inviscid magnetostrophic equations which attempt to implicitly preserve Taylor’s constraint as zero^35^,^36^.

Our conclusion on the potentially crucial role of the boundary conditions is quite opposite to a view held by some within the modelling community that the choice of boundary conditions does not matter. This view is summed up in Aubert^37^, (see also^14^), who writes, “It is generally believed that in the limit of vanishing Ekman number, the results of simulations with rigid and stress-free boundaries should converge.” This belief is founded on models that are located very far from the Earth’s core in parameter space. Indeed, the choice of model parameters, other than the Ekman number, for example the Prandtl number, *P*_*r*_, (which is O(10^−6^) in Earth’s core and typically O(1) in numerical models) is known to have a fundamental affect, at least at onset, on the role of boundary layers in non-magnetic convection. At high *P*_*r*_, although boundary layers are passive at the onset of non-axisymmetric flow, they play a role in the creation of the axisymmetric flow caused by nonlinear interactions. By contrast, at low *P*_*r*_, viscous boundary layers are always active, and the choice of boundary conditions then directly affects convective onset^18^. Our main conclusion here is that a degree of caution should be adopted for (quasi-) magnetostrophic models aiming to describe the dynamics of the core: the choice of boundary conditions may alter the geostrophic flow, a dominant part of the axisymmetric solution, in a fundamental fashion. Such concerns will not apply in three-dimensional models that exactly satisfy magnetostrophy, because weak viscosity is not required at all in the force balance. However, at the present time such models remain far out of reach and any departures from exact balance, either temporary or long-lived, drive large flows controlling not only the dynamics but the time-step for computational stability^10^.

An application of the results of this paper is in the links between outputs of numerical geodynamo models of the core to observations. Specifically, it has been demonstrated that zonal flows (as inferred from geomagnetic secular variation) are not only closely correlated with changes in the length-of-day^38^,^39^, but may be an important component in explaining the apparent westward drift of geomagnetic features near the equator^40^, possibly caused by advection^41^,^42^. In our models, the geostrophic flows were mainly positive, indicating eastward motion, although the choice of the driving Lorentz force was arbitrary and different examples (either kinematic or dynamic) would drive geostrophic flows with very different structures, for example, to be predominantly westward directed. As we showed in our example, for the same body force, the use of stress-free rather than no-slip conditions resulted not only in a significant change of overall magnitude of the geostrophic flow, but also a change in sign near the equator. Such associated changes in the structure of the zonal flow could fundamentally alter the ability of a model output to be linked to geophysical observations since, for example, the direction of the drift of geomagnetic flux patches due to the geostrophic flow may change from westwards to eastwards by simply using different boundary conditions. Such concerns are of direct importance to those involved in geomagnetic data assimilation^43^, whose ultimate goal is to match numerical models with geophysical observations. It is noteworthy that not all groups involved in this endeavour use the same mechanical boundary conditions: Kuang *et al.*^44^ and subsequent studies being based on^11^ have adopted stress-free boundaries, whereas Fournier *et al.*^45^, Li *et al.*^46^ and related studies use no-slip.

One further feature of our solutions is that the Ekman number needs to be smaller than 10^−5^ to get close to the asymptotically low-E regime (see also^47^). Values of *E* larger than this give solutions which are qualitatively unconverged. This means that, not only may the numerical models not correctly represent the physical processes of the core, but that any interpretation and links with geophysical observations may be difficult. For example, Fig. 5 demonstrates that the geostrophic flow at *E* = 10^−4^ is always positive (eastwards directed), whereas at Ekman numbers of 10^−5^ and below there is a significant westward flow over roughly a third of the domain. The internal shear caused by these oppositely directed jets would generate significant internal toroidal magnetic field and likely cause the dynamics of magnetic field generation to be fundamentally altered. It is probable therefore that the observational signature on both short and long-timescales of models at high *E*, such as reported in^48^ (using *E* ~ 10^−3^), may significantly differ from those obtained at more realistic values of *E*.

One issue with our model that may affect the role of the boundary conditions is the kinematic prescription of the body force, and the lack of any dynamical feedback. In a nonlinear time-dependent model, it may be that dynamical adjustments of the body force mean that the geostrophic flow becomes independent of boundary condition, contrary to our results presented. A suggestion of this behaviour can be found in^25^ who showed that the energy of an axisymmetric mean-field solution was apparently independent of boundary condition at asymptotically low-*E*, although they were unable to properly resolve the spatial structure of the geostrophic flow. Further studies, investigating the role of boundary conditions in not only weakly viscous but inviscid (2D) magnetostrophic models^35^ containing dynamic feedback, are a natural extension to this study and may serve to resolve this issue.

Lastly, we note some likely shortcomings of our model in describing the dynamics of the Earth’s core. Two structural effects, absent from our model yet likely present within the core are (i) surface topography on the underside of the core-mantle boundary, and (ii) stratification in the outermost outer core, both of which may have significant consequences. Surface topography is seismically inferred but the constraints from the available data are poor, typical values of several km are common^49^. However, given the approximate depth of the Ekman layer of only 1 m, this topography would completely alter the standard boundary layer dynamics, which is based on a smooth spherical surface^50^,^51^,^52^. Furthermore, any topography may itself be contained within a stably stratified layer of several hundred km at the very top of the core, of either thermal or chemical origin^53^. Such a layer may serve to disconnect the Ekman layer from the free-stream flow, leading to a partial or even full suppression of the boundary-driven zonal flows (e.g.^54^).

## Additional Information

**How to cite this article**: Livermore, P. W. *et al.* A comparison of no-slip, stress-free and inviscid models of rapidly rotating fluid in a spherical shell. *Sci. Rep.* **6**, 22812; doi: 10.1038/srep22812 (2016).
